# Development of an Innovative Pressurized Liquid Extraction Procedure by Response Surface Methodology to Recover Bioactive Compounds from Carao Tree Seeds

**DOI:** 10.3390/foods10020398

**Published:** 2021-02-11

**Authors:** Jhunior Abrahan Marcía Fuentes, Lucía López-Salas, Isabel Borrás-Linares, Miguel Navarro-Alarcón, Antonio Segura-Carretero, Jesús Lozano-Sánchez

**Affiliations:** 1 Faculty of Technological Sciences, Universidad Nacional de Agricultura, Catacamas, Olancho 16201, Honduras; juniorabrahamm@yahoo.com; 2 Faculty of Pharmacy and Food, University of Havana, La Lisa 17100, Havana, Cuba; 3 Department of Food Science and Nutrition, University of Granada, Campus Universitario s/n, 18071 Granada, Spain; lslucia@correo.ugr.es (L.L.-S.); nalarcon@ugr.es (M.N.-A.); jesusls@ugr.es (J.L.-S.); 4 Functional Food Research and Development Centre (CIDAF), Health Sciencie Technological Park, Avda. Del Conocimiento s/n, 18016 Granada, Spain; ansegura@ugr.es; 5 Department of Analytical Chemistry, Faculty of Sciences, University of Granada, 18071 Granada, Spain

**Keywords:** carao tree seeds, phenolics, PLE, HPLC-MS, bioactive compounds

## Abstract

Nowadays there are evidences from several studies which have revealed the protective effects of food against chronic diseases. These healthy properties have been related to bioactive compounds. Among bioactive substances, the scientific interest in phenolic compounds has stimulated multidisciplinary research on the composition of plant phenolic compounds. The aim of this work has been to determine the bioactive composition of Carao tree seeds (*Cassia grandis*) and to optimize the recovering of these compounds for developing functional ingredients. To achieve this goal, pressurized liquid extraction (PLE) has been applied to recover these phytochemicals. The optimization of this innovative extraction procedure was performed by a response surface methodology (RSM) based on a central composite design 2^3^ model to address the bioactive compounds extraction. Phenolic compounds recovered by PLE were characterized using reversed-phase high-performance liquid chromatography coupled to electrospray ionization time-of-flight mass spectrometry (HPLC-ESI-TOF-MS). Analytical characterization allowed the identification and quantitation of phenolic compounds belonging to hydroxybenzoic acids and flavonoids (flavonols, flavanols, flavanones and proanthocyanidins). Phytochemical concentrations were used as response variable in order to get the best extraction conditions. These results pointed out that Carao tree seeds can be a potential source of bioactive compounds and PLE extracts could be used as functional ingredients.

## 1. Introduction

Nowadays, the well-known relationship between diet—health has generated a lot of interest among current consumers, who are looking for healthy foods. In this sense, fruits and vegetables are highly appreciated for their health benefits, which are directly linked to the high number of biologically active components present in their composition. These substances, called bioactive phytochemicals or bioactive compounds, possess different nature and very diverse structures. Among these, phenolic compounds, which are considered secondary metabolites of plants, have increased the scientific interest in different areas [1,2]. Phenolic compounds can be classified into two different types: simple phenols and polyphenols. Phenolic acids (benzoic and cinnamic acids) and benzoquinones are part of the group of simple phenols, while polyphenols include flavonoids, stilbenes, lignans, tannins and other polymerized compounds. Regarding flavonoids, they deserve special attention due to their widely chemical groups: chalcones, dihydrochalcones, flavonols, dihydroflavonols, flavonoids (flavan-3-ols), flavones, flavanones, isoflavonoids and anthocyanidins. The bioactive properties that have been attributed to phenolic compounds are diverse, and their antioxidant capacity is the best-known effect [3,4]. In addition, other properties have been described such as anticancer, anti-inflammatory, antihypertensive, estrogenic, protective effects against cardiovascular disease, anti-hormone, antidiabetic and antithrombotic, among others [5,6].

Recovering of phenolic compounds from many parts of plants have been carried out to develop functional ingredients. To achieve this goal, new techniques have been introduced to improve the extraction process, such as pressurize liquid extraction (PLE). PLE allows to get natural extracts from plants combining elevated temperature and pressures with solvents to achieve fast and efficient extraction with a wide range of compounds polarities [7]. Indeed this technique has been applied to recover this kind of phytochemicals from different vegetables, fruits and oils as well as the by-products generated over the production process i.e., *Hibiscus sabdariffa* calyces, *Sclerocarya birrea* stem, pomegranate peel, weet cherry stem, and olive oil by-products [8,9,10,11,12,13,14]. In these studies, response surface methodology (RSM) was applied to optimize the phytochemical recovering, since its combination with advanced extraction technique may enhance the bioactive-extract production process. Consequently, RSM coupled to PLE provide a simply way to understand the extraction process as well as to reach the optimum conditions to increase the phenolic compounds extraction efficiency from vegetable matrices.

*Cassia grandis*, also known as “carao” and commonly called “pink shower”, is a tree of the *Fabaceae* family native from Central and South America. It is a medium-sized tree that matures to 15–30 m tall characterized by producing large pinnate leaves, a spectacular bloom with striking coral pink flowers that appear in early spring (hence its name “pink shower”) and large bean-shaped seed pods. After blooming, the flowers are replaced by thin woody pods that grow up to 2 and 3 inches long, containing between 70 and 80 seeds per pod. Inside the pods, the seeds are oriented transversely and separated into individual cells. In each partition there is a round, flattened, tan colored seed, which is surrounded by a dark brown, sticky, bittersweet pulp with a strong smell. For its shade, this tree is planted as an ornamental tree in gardens and avenues [15].

*C. grandis* is considered as food since its pods are edible for humans and in Central America the seed membrane, is used as a chocolate substitute. Scientific reports have pointed out the antioxidant properties of Carao seeds, which explains its use in traditional medicine [16]. Part of these functions could be related to its composition in bioactive compounds like simple phenols, flavonoids and tannins [16]. Indeed, different authors have reported the biological activity of phytochemicals belonging to these chemical groups [1,6]. Since scientific knowledge has pointed out that enriched extracts in this kind of phenolic compounds have interesting technological and pharmaceutical properties, they could be applied to develop food antioxidants or as ingredients in nutraceutical products.

The aim of this research was to analyze and optimize the extraction of the phenolic profile from *C. grandis* seeds through the combination of advanced extraction system and analytical platform. Response surface methodology was applied to obtain PLE enriched extracts which were characterized by high-performance liquid chromatography coupled to electrospray ionization time-of-flight mass spectrometry (HPLC-ESI-TOF-MS).

## 2. Materials and Methods

### 2.1. Reagents

All reagents used in this work were of analytical reagent grade. For extraction procedure, water used as solvent was purified by a Milli-Q system from Millipore (Bedford, MA, USA) and ethanol were purchased from VWR Chemicals (Radnor, PA, USA). Sand and cellulose filters were purchased from Fisher Chemicals (Waltham, MA, USA). For mobile phase preparation, formic acid was provided by Sigma-Aldrich (Steinheim, Germany) and LC-MS-grade acetonitrile was purchased from Fisher Chemicals (Waltham, MA, USA). The standards for the calibration curves (gallic acid, catechin, epicatechin, epigallocatechin-gallate, quercetn-3-glucoside and kaempferol-3-rutinoside) were acquired from Fluka, Sigma-Aldrich (Steinheim, Germany) or Extrasynthese (Genay Cedex, France).

### 2.2. Plant Material

Fresh samples of the carao fruit (*C. grandis*) were randomly collected in the wild with optimal maturity in the Guapinol Biological Reserve, Marcovia Municipality, Choluteca Department (Honduras), between February and March 2020. The total amount of the collected samples was 100 kg. Manual separation was carried out in different parts and the seeds were dried in an air circulation oven (Digitronic TFT- Selecta, J.P. SELECTA, Barcelona, Spain), during 48 h at 50 °C. Then, seeds were ground with a Retsch SM-100 brand blade mill (Retsch), equipped with a sieve from 501 to 700 μm, and vacuum packed. The processed samples were preserved at room temperature (30 °C ± 5 °C) until the extraction process.

### 2.3. Pressurized Liquid Extraction

Recovery of phytochemicals was carried out using a Dionex ASE 350 Accelerated Solvent Extractor (Dionex Corp., Sunnyvale, CA, USA). Extractions were done with different experimental combinations among solvent composition (ethanol and water), temperatures and extraction time. All extractions were carried out at constant pressure (11 MPa) and under a N_2_ atmosphere.

To carry out the extractions, the solvents were previously degassed for 15 min to remove the dissolved oxygen in order to avoid any possible oxidation. For each extraction, 3 g of sample were mixed with 9 g of sand and loaded onto 33 mL stainless-steel extraction cells. The chosen configuration was sandwich type (5 g sand + mixture sample − sand + 5 g sand). Cellulose filters were placed at each end of the cell in order to prevent clogging of the metal frits. The extraction conditions described above were applied and the obtained extracts were collected in glass vials. These extracts were quickly cooled to room temperature, filtered, and vacuum evaporated using a Savant SpeedVac Concentrator SC250EXP (Thermo Scientific, Sunnyvale, CA, USA) and stored at −20 °C until HPLC analysis.

### 2.4. Design of Experiments

Response surface methodology (RSM) was used to evaluate the effect of PLE parameters on retrieval and yield of phenolic compounds, using Statgraphics Centurion XV software version 15.1.02. The applied design model was a central composite design 2^3^ (CCD) model with two axial points and two levels (maximum and minimum) for each independent variable. Temperature, percentage of ethanol and extraction time were chosen for independent variables, and the experimental design consisted of a total of 14 experiments that were performed in a randomized order (Table 1). The experimental design covered the entire operational range of the temperature and solvent ratio that the device allows.

Response variables were the chemical composition of the extracts determined by HPLC-ESI-TOF-MS and yield. The extraction yield of each procedure was calculated considering the weight of dried extract and the amounts of carao seed used in the procedure (Equation (1)):

(1) $$\mathrm{Yield}\;\left(\%\right)=\frac{\mathrm{Weight}\;\mathrm{of}\;\mathrm{dried}\;\mathrm{extract}\;\left(g\right)}{\mathrm{Weight}\;\mathrm{of}\;\mathrm{dried}\;\mathrm{seeds}\;\mathrm{used}\;\left(g\right)}\;\times \;100$$

The obtained results were integrated into the experimental design with the Statgraphics Centurion 15.0 software. The adequacy of the model obtained for PLE were checked by evaluating coefficient of determination (*R*^2^), coefficient of variation (CV) and the Fisher’s test value (F-ratio). Significant values were considered when *p* < 0.05. Equations of the model adjusted for extraction yield and content in total phenolic compounds were obtained according to a second-order polynomial model. The 3D response surface plots allowed visualizing the relationship between independent variables and responses, representing the dependent variables in function of two most influent independent variables. Optimum conditions were calculated considering the maximization of individual response variables.

### 2.5. HPLC-ESI-TOF-MS Analysis

The PLE extracts were analyzed with an RRLC 1200 system (Agilent Technologies, Palo Alto, CA, USA), equipped with a vacuum degasser, a binary pump, an automated sampler, a thermostatic column compartment and a Diode Array Detector (DAD). The analytical column used for chromatographic separation was a 150 mm × 4.6 mm id, 1.8 μm particle diameter Zorbax Eclipse Plus C18 column (Agilent Technologies, Palo Alto, CA, USA). The mobile phase was 0.1% formic acid in water as eluent A and acetonitrile as eluent B. The injection volume was 10 μL. The flow rate of the mobile phase was 0.5 mL/min and the column temperature was maintained at 25 °C. Total run time was 45 min with a multi-step linear gradient applied for the phytochemical separation: 0 min, 5% B; 15 min, 65% B; 36 min, 95% B; 40 min, 5% B, and, then, a conditioning cycle of 5 min with the initial conditions before the next injection.

The HPLC system was coupled to an electro-spray time-of-flight mass spectrometer (HPLC-ESI-TOF-MS). The flow rate that reached the TOF-MS detector was 125 μL/min. Detection was performed in negative-ion mode over a range from 50 to 1000 *m/z.* The values of the source parameters were: capillary voltage of +4.5 kV; drying gas temperature, 190 °C; drying gas flow, 9 L min^−1^; and nebulizing gas pressure, 2.0 bar. The values of transfer parameters were: capillary exit, −150 V; skimmer 1, −50 V; hexapole 1, −23 V; RF hexapole, 100 Vpp; and skimmer 2, −22.5 V.

External mass-spectrometer calibration was performed using a 74900-00-05 Cole Palmer syringe pump (Vernon Hills, IL, USA) directly connected to the interface, equipped with a sodium formate solution 10 Mm. The mixture was injected at the beginning of each analysis and all spectra were calibrated before to the compounds identification. The exact mass data of the molecular ions were processed by Data Analysis 4.0 software (Bruker Daltonics, Billerica, MA, USA), which provided a list of possible elementary formulas using Generate-Molecular Formula Editor.

To carry out the identification and quantification of the analytes present in the PLE extracts, the samples were prepared at a concentration of 5 mg/mL using the hydro-alcoholic mixture of ethanol and water (50:50, v:v) as solvent. Each extract was analyzed in triplicate. To carry out the quantitative analysis, calibration curves of the six standards described in the reagents section were prepared at a concentration of 1 mg/mL. In order to draw the calibration curve for each standard compound, dilutions were prepared and analyzed at the following concentrations: 0.5; 1; 2.5; 10; 20; 30; 50; 75; 100 and 150 ug/mL.

## 3. Results

### 3.1. Identification of Polar Compounds in PLE Extracts of C. grandis by HPLC-ESI-TOF-MS

The compounds were identified by the data provided by HPLC-ESI-TOF-MS. The mass spectra obtained for the different PLE extracts were compared with the spectra of the available standards, so it was possible to identify some of the compounds present in the sample. All other compounds for which commercial standards were not available were identified by DataAnalysis 4.0 software. This software provided a list of possible elementary formulas by using the Generate-Molecular Formula Editor. The search of the formulas generated in the literature made it possible to identify a large number of chromatographic peaks obtained in the HPLC-MS chromatogram.

Most of the studies used in identifying these compounds were those that evaluated the chemical composition of other species of the genus *Cassia*, Fabaceae family or specimens of the same *C. grandis* class [9,10,11,12,13,14,15,16,17,18,19,20,21,22].

**Figure 1:**
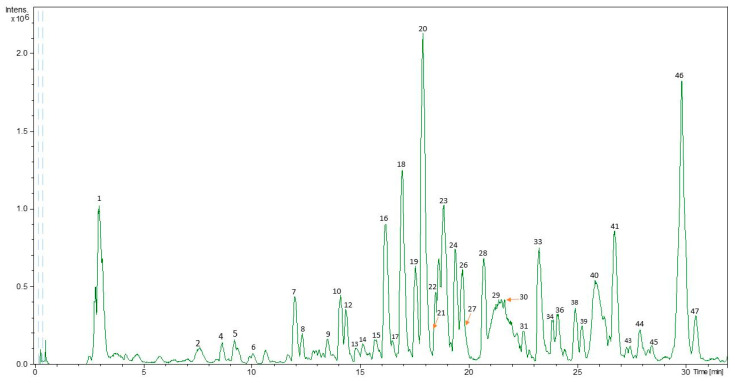
Base peak chromatogram (BPC) of representative pressurized liquid extraction (PLE) extract obtained by high-performance liquid chromatography coupled to electrospray ionization time-of-flight mass spectrometry (HPLC-ESI-TOF-MS).

Figure 1 showed a base peak chromatogram (BPC) representative of the carao seed extracts obtained by HPLC-ESI-TOF-MS in negative polarity. A total of 47 compounds were detected and included in Table 2 that summarized the following information: retention time, experimental and theoretical *m/z*, error (ppm), mSigma, molecular formula, a list of proposed compounds as well as the extraction conditions in which the compounds were characterized. The identified compounds were tentatively identified as disaccharides, hydroxybenzoic acids, flavonoids, steroids and quinones. The mass spectra of these compounds are shown in Supplementary Figures S1 and S2. Despite that the information reported by the analysis of the extracts, it was not enough to the identification of some of these peaks, which being listed as unknown.

#### 3.1.1. Disaccharides

Peak 1, with *m/z* 341 and molecular formula C_12_H_22_O_11_, was identified as sucrose. Indeed, this compound has previously been described in other studies about chemical characterization of fruits such as cocoa and grapes [17]. Since the analyzes were developed in reverse phase, this type of compound being more polar than others, eluded at the beginning of the chromatogram.

#### 3.1.2. Hydroxybenzoic Acids

The examination of mass spectra and elution profile of compounds in *C. grandis* seeds revealed two hydroxybenzoic acids (Peaks 2 and 3), respectively identified as galloyl glucoside and its derivative, according to the literature [18]. These compounds were previously described in *Sclerocarya birrea*, which belongs to the same division (magnoliophyta) and class (magnoliopsida) of *C. grandis*.

#### 3.1.3. Flavonoids

Compounds belonging to this chemical group were the major identified phenolics. Indeed, this family has widely been described in other *Cassia* families [19]. In this particular case, within this group, four different subclasses of compounds have been detected: flavonols, flavanols, flavanones and proanthocyanidins, in addition to a derivative of flavones.

The flavanols subclass included Peaks 17, 27, 30, 36 and 38. Peak 17, at retention time of 16.47 min, which displayed *m/z* at 449, was detected in all extraction conditions. It was identified as astilbin, a compound also described in *Cassia bakeriana* [20]. Peak 27 was detected in all conditions except to PLE 2, and it was determined to be quercetin-3-glucoside, being confirmed with the commercial standard. In the case of Peak 30, *m/z* 447 and molecular formula C_21_H_20_O_11_, it was identified as quercetin-rhamnoside. This chemical compound was also identified in *Cassia abbreviate* [19]. Peaks 36 and 38, both with *m/z* 431 and the same molecular formula C_21_H_20_O_10_, were proposed as kaempferol-rhamnoside or isomer [20].

Peaks 9, 11, 16, 22, 25, 40, 41, 44, 45 and 47 were characterized as flavanols. Peak 9, which was recovered under all extraction conditions, was proposed as theaflavin [19]. Peaks 11 and 16 were identified, respectively, as catechin and (epi)-catechin [19], and these identifications were also confirmed thanks to commercial standards. Peaks 22 and 25 showed the same mass spectrum, generating the same molecular formula and being identified as (epi)-afzelechin or isomer [19]. However, it was important to remark that Peak 22 appeared in all extraction conditions, but Peak 25 was only detected in PLE 11 and PLE 12. Peaks 40, 41, 44, 45 and 47, gave the same *m/z* at 513 and generated the same formula (C_30_H_26_O_8_), being identified as diflavanoid or isomer. These chemical compounds were reported as flavanol derivatives [21]. 

With regard to flavanones, Peaks 18 and 20 were characterized as pinocembrin-7-neohesperidoside and pinocembrin-7-rutinoside, respectively [22,23,24,25]. Their chemical structure was a flavanone (pinocembrin) linked to two different disaccharides (neohesperidoside and rutinoside). These compounds were characterized in the species *Litchi chinensis*, *Euphorbia decipiens* and *Ziziphora clinopodioides*, which belong to the same division and to the same *C. grandis* class.

Peak 46, with retention time of 29.83 min and *m/z* 401, was identified as hexamethoxyflavone, a derivative of flavones. This compound has been described in *Citrus* and *Murraya* [26,27,28], which belong to the same division and class of *C. grandis*.

Finally, the subclass of proanthocyanidins included Peaks 8, 12, 13, 14, 21, 23, 29, 31, 32, 33, 34, 35, 37 and 39. Peaks 8 and 12 were detected in all extraction conditions. Their spectra provided the same *m/z* and molecular formula, being characterized as (epi)-gallocatechin-(epi)-catechin (prodelphinidin B3) or its isomers according to the literature [18]. Peak 13 with *m/z* 579 and molecular formula C_30_H_27_O_12_, was proposed as procyanidin derivative, which was previously described in *Cassia fistula* [29]. Peak 14, which was present in all extraction conditions except to PLE 12 and PLE 14, was identified as (epi)-catechin-(epi)-catechin (proanthocyanidin B2) according to the literature [19]. Peak 21 was also detected in all extraction conditions and it was proposed as cassanidin A [19]. Peaks 23, 31 and 34 were identified as (epi)-guibourtinidol-(epi)-catechin or its isomers, and Peaks 29, 32, 33, 35 and 37 as (epi)-guibourtinidol-(epi)-afzelechin or its isomers [19]. The last of the phenolic compounds (Peak 39) was identified as catechin-guibourtinidol-cassiaflavan [19].

#### 3.1.4. Other Polar Compounds

In relation to other non-phenolic polar compounds, Peak 27, within steroids, was characterized as physalin A [30]. These authors reported the presence of this compound in *Physalis alkekengi*, which belongs to the magnoliophyta division and to the magnoliopsida class as well as *C. grandis.* Finally, quinones family included Peak 41, at *m/z* 253 and molecular formula C_15_H_10_O_4_. It was identified as chrysophanol, previously described in *Cassia tora* [31].

### 3.2. Quantification of Polar Compounds in C. grandis Seed PLE Extracts by HPLC-ESI-TOF-MS

In order to quantify the amount of polar compounds present in *C. grandis* seed, six calibration curves were prepared using gallic acid, catechin, epi-catechin, epigallocatechin-gallate, quercetin-3-glucoside and kaempferol-rutinoside (Table 3). Calibration curves showed good linearity between the different concentration ranges depending on the analyte studied. In all cases, the linearity of calibration curves was better than 0.99. The concentration of the phenolic compounds present in the extracts was calculated using the individual area obtained by HPLC-ESI-TOF-MS analyzes of each compound and interpolating in the corresponding calibration curve. For this purpose, the calibration curves of the available commercial standard or those with a similar structure were used for each phenolic compound. Hydroxybenzoic acids were quantified using the gallic acid calibration curve. Catechin standard was used to quantify this compound as well as the rest of the flavanols present in the extracts. (Epi)-catechin was quantified with its own commercial standard. Theaflavin derivative, as well as the flavanones pinocembrin-7-neohesperidoside and pinocembrin-7-rutinoside, were quantified with kaempferol-rutinoside.

Within flavonols, quercetin-3-glucoside and quercetin-rhamnoside were quantified with the quercetin-3-glucoside calibration curve. Kaempferol-rhamnoside was quantified with the standard kaempferol-rutinoside.

The derivative of flavones (hexamethoxyflavone) had a structure similar to catechin, with the difference that its hydroxyl groups were methylated. Due to this similarity, it was quantified with this commercial standard. The same calibration curve was used to quantify the diflavanoids present in the extracts. Finally, all the proanthocyanidins were quantified using the epigallocatechin-gallate calibration curve.

Table 4 showed the yield and the total polar compound for all PLE extracts. With regard to the yield, these results pointed out that these values were within those described for this technique when it was applied to other plant matrices [7,32]. The extraction conditions with the highest yield were PLE 14 (200 °C, 50% EtOH, 12.5 min), PLE 9 (110 °C, 50% EtOH, 12.5 min) and PLE 8 (110 °C, 50% EtOH, 12.5 min). The extraction conditions PLE 8 and PLE 9 were the central points of the design by presenting the same values of the independent variables. For both conditions, the results were similar (28.45% and 29.91%, respectively), concluding the reproducibility of the extraction conditions.

Based on the results obtained, it can be noted that in general, the application of elevated temperatures above 100 °C combined with percentages equal to or greater than 50% of ethanol and long times (12 min) resulted in higher yields. In effect, the diffusivity of the solvent increases with increasing temperature, as it has been described in the literature [33]. However, it was important to consider that an increase in temperature can affect to thermolabile compounds such as phenolic compounds [32].

The total phenolic content (Table 4) and total hydroxybenzoic acids, flavanols, flavonols, flavanones, flavones, and proanthocyanidins/prodelphinidisns (Table 5 and Table 6) were estimated as the sum of the individual phenolic compound belonging to each family. These concentrations were expressed in mg analyte/g extract. The range of total phenolic content obtained under different PLE conditions was similar to those described in the literature for plant phenolic extracts obtained by advanced extraction techniques [34,35]. 

As it can be seen, PLE 1 condition (40 °C, 15% EtOH, 20 min) was the best combination in the extraction of hydroxybenzoic acid family. Flavanol, flavone and flavanone families were extracted in greater amount with the conditions applied in PLE 10 (40 °C, 85% EtOH, 20 min). Flavanols had high concentrations in almost all extraction conditions, as did proanthocyanidins, although in the latter, the highest concentration reached in the PLE 7 extract (110 °C, 50% EtOH, 3 min) could be highlighted.

### 3.3. PLE Extraction Design Optimization

Extraction yields and total phenolic compounds values obtained by PLE conditions were integrated into the experimental design with Statgraphics Centurion 15.0 software. Table 7 shows the analysis of the proposed model showing linear, quadratic and interactions effects among independent variables (X_1_:Temperature, X_2_:EtOH and X_3_:Extration time) on the variable responses extraction yield (Y_1_) and total phenolic compounds (Y_2_).

The analysis of independent variables was adjusted to a 95% of confidence level, so the value of *R*^2^ for each of the variables indicated that the model, thus adjusted, explained 95.0% and 96.6% of the variability in yield and total phenolic compounds variables, respectively. To verify if the selected model was adequate to explain the observed data, or if a more complicated model should be used, the lack-of-fit test was also included. Since the *p* value for the lack-of-fit in the ANOVA table was greater than 0.05 for both variables, the model seemed to be adequate for the data observed at a confidence level of 95.0%. The significant effects of each independent variable were those which had a *p*-value equal to or less than 0.05. Therefore, for those variables whose quadratic effects or interactions with other variables did not present significant effects, they were eliminated from the model’s adjustment equation [7].

The fitted equations of the model for each response variables are described below (Equation (2)):

(2) $$Y1=\;-13.7306+0.108894\;X_{1\;}+0.931317\;X_{2\;}+0.141351\;X_{3}-0.00933134\;X_{2}^{\;2}\;Y2=\;-128.608+3.97862\;X_{1}+5.54721\;X_{2}+11.4992\;X_{3}-0.0162721\;X_{1}^{\;2}-0.112334\;X_{1}X_{3}-0.0376946\;X_{2}^{\;2}$$

Equation (2): Equations of the fitted model: (a) extraction yield (Y1); and (b) content of total phenolic compounds (Y2).

Concerning yield, the main variables that affected this response were temperature and the quadratic effect of the percentage of ethanol used in the extraction. With regard to the phenolic compounds, temperature and ethanol were statistically significant. However, in relation to the quadratic effects and the interactions between independent variables, the response variable of total phenolic compounds was significantly affected by the quadratic effects of temperature and ethanol, as well as the interactions of temperature with extraction time.

**Figure 2:**
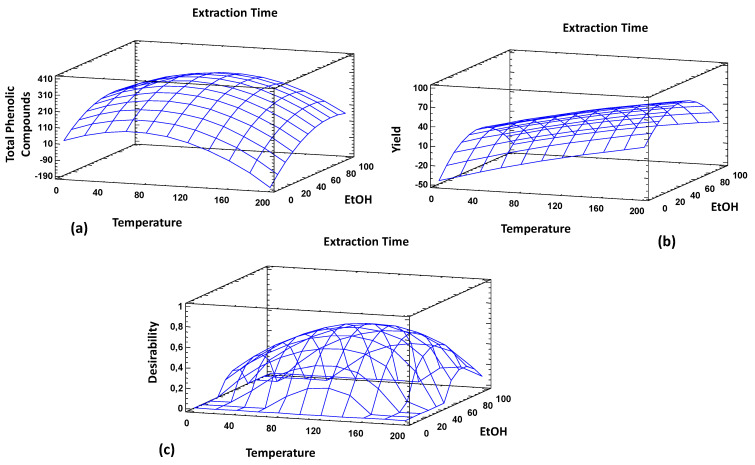
Surface response graphs: (**a**) phenolic compounds; (**b**) extraction yield; and (**c**) optimization of multiple responses.

Figure 2 showed the individual and multiple response surface plots.

Finally, Table 8 included the predictable results according to the optimum conditions provided by the model for each of the independent variables.

The theoretical-optimum yield value established by the experimental design determined that to maximize the extraction yield, the following PLE conditions could be applied: 49.8% ethanol, 200 °C, and an extraction time of 22 min. The proposed ethanol percentage was within the experimental region of the model. The PLE extractions carried out at the laboratory reported that the highest yield values were obtained with PLE 8, PLE9 and PLE14 conditions (Table 1 and Table 4). In all of these conditions, 50% ethanol was combined with high temperatures, up to 200 °C in the highest yield condition (29.97%), being this value the operational limit of the PLE device. This temperature corresponds to the maximum value proposed by the model to maximize the extraction yield. It is important to remark that this variable was tested in its fully operational range allowed by the device, and consequently, in a practical application it would be not possible to increase the maximum value established by the proposed design. With respect to the extraction time, its theoretical optimum value to maximize extraction yield could be obtained with a total run time of 22 min in combination with the other factors. Since this value was the maximum time applied in the experimental runs, it could indicate that the model did not cover all experimental region for this independent variable. However, it could be taken into account that extraction time effects were not significant to this response variable (Table 7). Indeed, the experimental results pointed out that the extraction yield obtained for PLE4 and PLE7 were similar, 19.9 and 20.19%, respectively. These experimental runs were carried out at the same temperature (110 °C) and percentage of ethanol (50%), but different extraction times: 22 min (PLE4) and 3 min (PLE7). In addition to that, reduction of the extraction time from 22 min (PLE4) to 12.5 min (PLE8 and PLE9) and keeping constant the rest of the experimental variables increased the extraction yield from 19.8 to 28.45–29.91% (PLE8 and PLE9, respectively).

Concerning the total phenolic compounds, the theoretical factors proposed to maximize this response variable were 46.3 °C, 73.8% ethanol and an extraction time of 22 min (Table 8). These proposed conditions were similar to the PLE 10 extraction condition: 40 °C, 85% ethanol and an extraction time of 20 min, for which a response variable value of 349 mg/g of extract was obtained. This value was similar to the theoretical optimum proposed by the model: 363 mg/g of extract. Analysing the independent variables, both of the theoretical values proposed for temperature and percentage of ethanol were within the experimental region evaluated by the proposed model. As it has been described above, temperature as well as percentage of ethanol showed significant effect in the recovery of this kind of phytochemicals by PLE. On the other hand, the better extraction time to maximize this response seems to be in the limit of the experimental region. Statistical analysis of the design showed that the extraction time was not significant for the phenolic compounds recovery by PLE. In fact, the analysis of the total phenolic content obtained in the experimental runs showed an independent behaviour with respect to the extraction time. For example, PLE4, PLE7, PLE8 and PLE9 were developed at 110 °C and 50% EtOH, being the total run time 22, 3, 12.5 and 12.5 min, respectively. All of these conditions provided similar recovery of bioactive compounds (Table 4).

Despite that, extraction time was not significant for both response variables, extraction yield and phenolic compounds did not showed correlation over the PLE experimental conditions evaluated. Therefore, the response variables showed different optimal PLE factors to maximize their recovery from Carao seeds. The main difference was concerning to the temperature values. The analyzed results proposed 200 °C and 46.3 °C to maximize the extraction yield and total phenolic content, respectively. Indeed the lower temperature to be applied for the recovering of phenolic compounds could be justify due to it is well-known that an increase in temperature can affect to thermolabile compounds such as phenolic compounds [32]. In order to maximize both responses, a multiple response analysis was carried out. The results pointed out that the theoretical values of extraction yield and phenolic compounds were minor that those proposed by the individual analysis.

## 4. Conclusions

The proposed extraction system using PLE allowed to obtain 14 extracts under different extraction conditions delimited by the technical limits of the PLE extractor. Chemical characterization of these extracts allowed detecting 47 compounds. The HPLC-ESI-TOF-MS analytical platform made it possible to identify these compounds, using the information provided by the mass spectrometer, as well as the scientific literature. In the same way, the proposed analytical methodology allowed the quantification of the identified phenolic compounds, belonging to the families of hydroxybenzoic acids, flavonoids (flavonols, flavanols, flavanones and proanthocyanidins) in addition to a derivative of flavones. Finally, the proposed surface response model pointed out the effect of the independent variables (temperature, percentage of ethanol and time) on the response variables: yield and total phenolic compounds. Statistical analysis of the results confirmed that the proposed model is appropriate for explaining the obtained results and allowed estimating the optimal theoretical values for each of the response variables based on optimal extraction conditions. An extraction process is feasible to the functional food industry when the process accomplished the extraction of bioactive compounds and reasonable amounts allowing the minimum solvent, time and energy consumptions. This methodology could be useful for producers to obtain phenolic compounds-enriched ingredients. The combination of this methodology with stabilization and formulation techniques could be applied to obtain functional ingredient with application to food antioxidants as well as to nutraceutical products.

## Figures and Tables

**Table 1:** Central composite model 2^3^. Values of independent factors.

Experimental Condition	Temperature (°C)	%EtOH	Static Cycle (min)
PLE 1	40	15	20
PLE 2	40	85	5
PLE 3	110	5	12.5
PLE 4	110	50	22
PLE 5	40	15	5
PLE 6	20	50	12.5
PLE 7	110	50	3
PLE 8	110	50	12.5
PLE 9	110	50	12.5
PLE 10	40	85	20
PLE 11	180	85	5
PLE 12	180	85	20
PLE 13	110	95	12.5
PLE 14	200	50	12.5

Temperature levels (°C): −α, α (20, 200), −1,1 (40,180) and 0 (110). EtOH levels (%): −α, α (5, 95), −1,1 (15, 85) and 0 (50). Static cycle levels (min): −α, α (3, 22), −1,1 (5, 20) and 0 (12.5).

**Table 2:** Phenolic and other polar compounds of *C. grandis* PLE extracts characterized by HPLC-ESI-TOF-MS.

Peak	RT (min)	Proposed Compound	*m/z*	*m/z Exp*	Molecular Formula	Error (ppm)	mSigma	PLE
1	2.96	Sucrose	341.1089	341.1171	C_12_H_22_O_11_	−6.1	36.4	*
2	7.59	Galloyl glucoside	331.0671	331.0696	C_13_H_16_O_10_	2.0	11.8	*
3	8.40	Galloyl glucoside derivative	315.0722	315.0675	C_13_H_16_O_9_	3.3	5.7	1, 3, 4, 5, 6, 7, 8, 11
4	8.66	UK1	397.1715	397.1736	C_16_H_29_O_11_	−4.1	13.3	2, 10
5	9.20	UK2	380.1562	380.1574	C_15_H_26_NO_10_	−0.5	6.0	2, 10
6	10.05	UK3	371.0925	371.0993	C_23_H_16_O_5_	−13.4	32.6	1, 2, 3, 4, 5, 6, 7, 8, 9, 10, 11, 12, 13
7	11.99	UK4	443.1923	443.1963	C_21_H_32_O_10_	−8.8	3.6	*
8	12.33	(Epi)gallocatechin–(epi)catechin or isomer 1	593.1301	593.1511	C_30_H_26_O_13_	−19.6	5.1	*
9	13.48	Theaflavin derivative	771.2353	771.2391	C_34_H_44_O_20_	5.8	3.9	*
10	14.10	UK5	541.2173	541.2179	C_40_H_29_O_2_	6.5	72.4	*
11	14.23	Catechin	289.0718	289.0734	C_15_H_14_O_6_	−9.6	9.5	3, 4, 5, 8, 9, 11, 12, 14
12	14.33	(Epi)gallocatechin–(epi)catechin or isomer 2	593.1301	593.1523	C_30_H_26_O_13_	−18.9	28.4	*
13	14.82	Procyanidin derivative	579.1508	579.1728	C_30_H_27_O_12_	−19.5	11.8	1, 2, 3, 4, 7, 8, 9, 10, 12, 13
14	15.12	(Epi)catechin–(epi)catechin	577.1351	577.1368	C_30_H_26_O_12_	5.8	8.4	1, 2, 3, 4, 5, 6, 7, 8, 9, 10, 11, 13
15	15.72	UK6	401.1089	401.1096	C_17_H_21_O_11_	−1.9	10.6	1, 2, 3, 5, 6, 7, 8, 9, 10, 11, 12, 13
16	16.17	(Epi)-catechin	289.0718	289.0786	C_15_H_14_O_6_	−10.3	2.1	*
17	16.47	Astilbin	449.1089	449.1109	C_21_H_22_O_11_	2.9	6.4	*
18	16.94	Pinocembrin 7-neohesperidoside	563.1770	563.1743	C_27_H_32_O_13_	5.3	14.1	*
19	17.54	UK7	563.1864	563.1822	C_38_H_28_O_5_	7.3	10.6	*
20	17.91	Pinocembrin 7-rutinoside	563.1770	563.1778	C_27_H_32_O_13_	−14.5	12.8	*
21	18.41	(Epi)-afzelechin or isomer 1	273.0768	273.0777	C_15_H_14_O_5_	−1.8	36.9	*
22	18.50	Cassanidin A	817.2138	817.2133	C_45_H_38_O_15_	6.8	9.8	*
23	18.85	(Epi)-guibourtinidol-(epi)-catechin or isomer 1	545.1453	545.1547	C_30_H_26_O_10_	−13.1	40.4	*
24	19.32	UK8 or isomer 1	553.2232	553.2200	C_34_H_33_O_7_	2.4	6.6	2, 3, 5, 6, 10
25	19.37	(Epi)-afzelechin or isomer 2	273.0768	273.0840	C_15_H_14_O_5_	−15.8	7.2	11, 12
26	19.70	UK8 or isomer 2	553.2232	553.2195	C_34_H_33_O_7_	4.9	9.8	2, 3, 6, 10
27	19.77	Quercentin-3-glucoside	463.0882	463.0894	C_21_H_20_O_12_	−2.1	34.0	1, 3, 4, 5, 6, 7, 8, 9, 10, 11, 12, 13, 14
28	20.67	Physalin A	525.1766	525.1672	C_28_H_30_O_10_	17.0	17.3	*
29	21.46	(Epi)-guibourtinidol-(epi)-afzelechin or isomer 1	529.1504	529.1534	C_30_H_26_O_9_	−0.2	42.5	*
30	21.62	Quercetin- rhamnoside	447.0933	447.0951	C_21_H_20_O_11_	−7.7	38.6	*
31	22.49	(Epi)-guibourtinidol-(epi)-catechin or isomer 2	545.1453	545.1465	C_30_H_26_O_10_	9.0	10.0	1, 2, 4, 5, 6, 7, 8, 9, 10, 11, 12, 13, 14
32	22.56	(Epi)-guibourtinidol-(epi)-afzelechin or isomer 2	529.1504	529.1559	C_30_H_26_O_9_	−4.7	9.1	3, 12
33	23.20	(Epi)-guibourtinidol-(epi)-afzelechin or isomer 3	529.1504	529.1574	C_30_H_26_O_9_	−6.5	29.5	*
34	23.81	(Epi)-guibourtinidol-(epi)-catechin or isomer 3	545.1453	545.1470	C_30_H_26_O_10_	6.6	3.8	1, 2, 3, 4, 5, 6, 7, 8, 9, 10, 11, 13
35	23.96	(Epi)-guibourtinidol-(epi)-afzelechin or isomer 4	529.1504	529.1501	C_30_H_26_O_9_	7.3	34.8	1, 2, 3, 4, 5, 7, 8, 9, 11, 12, 13, 14
36	24.07	Kaempferol- rhamnoside or isomer 1	431.0984	431.0999	C_21_H_20_O_10_	−3.5	19.9	1, 2, 3, 6, 10, 11, 12, 13, 14
37	24.35	(Epi)-guibourtinidol-(epi)-afzelechin or isomer 5	529.1504	529.1498	C_30_H_26_O_9_	1.2	29.1	1, 2, 4, 6, 7, 8, 9, 10, 12, 13
38	24.87	Kaempferol- rhamnoside or isomer 2	431.0984	431.1004	C_21_H_20_O_10_	9.7	6.1	1, 2, 4, 5, 6, 7, 8, 9, 10, 11, 12, 13, 14
39	25.19	Catechin-guibourtinidol-cassiaflavan	785.2240	785.2271	C_45_H_38_O_13_	7.4	3.2	*
40	25.80	Diflavanoid or isomer 1	513.1555	513.1599	C_30_H_26_O_8_	−0.4	22.4	*
41	26.67	Diflavanoid or isomer 2	513.1555	513.1619	C_30_H_26_O_8_	−13.1	23.3	*
42	27.08	Chrysophanol	253.0506	253.0521	C_15_H_10_O_4_	−8.4	5.4	3, 5
43	27.39	UK9	697.2138	697.2121	C_35_H_38_O_15_	9.2	6.8	1, 2, 3, 4, 5, 6, 7, 8, 9, 10, 11, 12, 13
44	27.81	Diflavanoid or isomer 3	513.1555	513.1552	C_30_H_26_O_8_	6.7	16.5	*
45	28.24	Diflavanoid or isomer 4	513.1555	513.1541	C_30_H_26_O_8_	−11.4	21.9	1, 2, 3, 4, 5, 6, 7, 8, 9, 10, 11, 12, 14
46	29.82	Hexametoxyflavone	401.1242	401.1180	C_21_H_22_O_8_	12.5	16.8	*
47	30.44	Diflavanoid or isomer 5	513.1555	513.1556	C_30_H_26_O_8_	−0.3	12.3	*

RT: retention time. * Indicates that they were identified in all the extracts. For those compounds that were not identified in all the extracts, the number of the extract in which they were identified was indicated. UK, unknown.

**Table 3:** Calibration curves used in the quantification of phenolic compounds.

Pattern	Calibration Range (mg/L)	Calibration Curve	*R* ^2^
Gallic acid	1–150	y = 23,395x − 37,644	0.9926
Catechin	0.5 -20	y = 262,318x + 23,166	0.9963
Epi-catechin	0.5–50	y = 287,543x + 575,127	0.9925
Epigallocatechin-gallate	0.5–150	y = 82,849x + 198,670	0.9918
Quercetin-3-glucoside	0.5–20	y = 457,785x + 340,216	0.9916
Kaempferol-rutinoside	0.5–150	y = 115,620x + 754,772	0.996

**Table 4:** Extraction yield and quantitative results of PLE central composite 2^3^ experimental design expressed in mg compound/g extract of *C. grandis* seed (X ± SD).

Experimental Design Condition	Yield (%)	Total Polar Compound (mg Compound/g Extract)
PLE 1	4.44	262 ± 4
PLE 2	3.82	236 ± 1
PLE 3	5.16	87 ± 4
PLE 4	19.9	266 ± 3
PLE 5	3.14	136 ± 1
PLE 6	11.15	200 ± 2
PLE 7	20.19	327 ± 5
PLE 8	28.45	286 ± 5
PLE 9	29.91	279 ± 2
PLE 10	4.82	349 ± 2
PLE 11	15.68	217 ± 7
PLE 12	23.51	114 ± 2
PLE 13	3.64	271 ± 12
PLE 14	29.97	46.8 ± 0.6

**Table 5:** Concentration of phenolic compounds in PLE extracts, ordered by families and expressed in mg compound/g extract (X ± SD).

	PLE 1	PLE 2	PLE 3	PLE 4	PLE 5	PLE 6	PLE 7
**Hydroxybenzoic Acids**							
Galloyl glucoside	25 ± 5	11.1 ± 0.6	11.7 ± 0.7	13.4 ± 0.9	16.1 ± 0.4	14 ± 1	16 ± 1
Galloyl glucoside derivative	2.2 ± 0.1	ND	2.36 ± 0.05	1.8 ± 0.1	3.4 ± 0.1	1.7 ± 0.3	2.1 ± 0.3
**Total hydroxybenzoic acids**	27 ± 5	11.1 ± 0.6	14 ± 1	15.1 ± 0.9	19.6 ± 0.4	15 ± 1	18 ± 1
**Flavanols**							
Theaflavin derivative	0.72 ± 0.02	1.12 ± 0.03	0.163 ± 0.005	0.86 ± 0.04	0.249 ± 0.004	0.69 ± 0.02	1.03 ± 0.02
Catechin	ND	ND	1.01 ± 0.09	2.3 ± 0.1	1.25 ± 0.03	ND	ND
(Epi)-catechin	8.3 ± 0.5	6.63 ± 0.05	2.1 ± 0.2	8.0 ± 0.4	3.0 ± 0.1	5.9 ± 0.3	8.4 ± 0.1
(Epi)-afzelechin or isomer 1	3.2 ± 0.5	2.1 ± 0.2	1.77 ± 0.06	4.0 ± 0.1	2.4 ± 0.1	2.2 ± 0.1	4.7 ± 0.2
(Epi)-afzelechin or isomer 2	ND	ND	ND	ND	ND	ND	ND
Diflavanoid or isomer 1	14 ± 1	11.4± 0.4	3.0 ± 0.1	15.9 ± 0.6	6.2 ± 0.2	11.8 ± 0.3	10.3 ± 0.2
Diflavanoid or isomer 2	11.5 ± 0.5	8.2 ± 0.4	1.94 ± 0.02	9.35 ± 0.03	3.1 ± 0.1	8.1 ± 0.4	13.6 ± 0.1
Diflavanoid or isomer 3	1.41 ± 0.06	1.09 ± 0.08	0.78 ± 0.02	3.3 ± 0.2	1.26 ± 0.05	1.04 ± 0.02	2.54 ± 0.06
Diflavanoid or isomer 4	0.18 ± 0.02	0.22 ± 0.01	0.197 ± 0.003	0.99 ± 0.08	0.23 ± 0.01	0.20 ± 0.01	0.476 ± 0.006
Diflavanoid or isomer 5	2.01 ± 0.07	1.80 ± 0.04	0.236 ± 0.006	1.78 ± 0.06	0.404 ± 0.004	1.98 ± 0.06	4.1 ± 0.1
**Total flavanols**	41 ± 1	33 ± 1	11.2 ± 0.3	46 ± 1	18.1 ± 0.4	31.8 ± 0.9	45.2 ± 0.3
**Flavonols**							
Astilbin	0.69 ± 0.04	0.30 ± 0.03	0.066 ± 0.004	0.70 ± 0.05	0.18 ± 0.01	0.30 ± 0.02	1.08 ± 0.05
Quercentin-3-glucoside	1.86 ± 0.08	ND	0.29 ± 0.02	2.03 ± 0.06	0.49 ± 0.03	0.66 ± 0.01	2.71 ± 0.1
Quercetin-rhamnoside	2.1 ± 0.2	0.91 ± 0.05	0.37 ± 0.01	2.07 ± 0.09	0.91 ± 0.05	1.23 ± 0.09	2.8 ± 0.1
Kaempferol-rhamnoside or isomer 1	1.8 ± 0.1	2.74 ± 0.05	0.26 ± 0.01	ND	ND	1.9 ± 0.1	ND
Kaempferol-rhamnoside or isomer 2	2.5 ± 0.2	2.5 ± 0.2	ND	1.13 ± 0.06	0.56 ± 0.02	2.2 ± 0.1	2.10 ± 0.06
**Total flavonols**	9.0 ± 0.3	6.5 ± 0.2	1.00 ± 0.02	5.94 ± 0.07	2.15 ± 0.03	6.3 ± 0.2	8.7 ± 0.2
**Flavanones**							
Pinocembrin 7-neohesperidoside	18 ± 2	24.9 ± 0.4	3.7 ± 0.2	15.1 ± 0.2	5.7 ± 0.2	13.7 ± 0.3	19 ± 1
Pinocembrin 7-rutinoside	25 ± 1	35.9 ± 0.9	7.7 ± 0.1	20.8 ± 0.6	13.5 ± 0.5	20.5 ± 0.4	26 ± 1
**Total flavanones**	43 ± 3	61 ± 1	11.4 ± 0.3	36.0 ± 0.7	19.1 ± 0.7	34.3 ± 0.6	45 ± 2
**Flavones**							
Hexametoxyflavone	2.9 ± 0.1	18.4 ± 0.8	1.38 ± 0.09	1.55 ± 0.04	2.8 ± 0.1	9.1 ± 0.3	3.00 ± 0.08
**Total flavones**	2.9 ± 0.1	18.4 ± 0.8	1.38 ± 0.09	1.55 ± 0.04	2.8 ± 0.1	9.1 ± 0.3	3.00 ± 0.08
**Proanthocyanidins/Prodelphinidins**							
(Epi)gallocatechin–(epi)catechin or isomer 1	2.7 ± 0.1	3.1 ± 0.2	0.52 ± 0.02	1.74 ± 0.03	0.80 ± 0.04	1.47 ± 0.09	2.30 ± 0.02
(Epi)gallocatechin–(epi)catechin or isomer 2	4.7 ± 0.5	6.2 ± 0.2	0.89 ± 0.03	4.2 ± 0.2	1.56 ± 0.02	3.8 ± 0.3	4.9 ± 0.1
Procyanidin derivative	1.7 ± 0.1	1.9 ± 0.1	0.62 ± 0.02	2.5 ± 0.1	ND	ND	2.2 ± 0.2
(Epi)catechin–(epi)catechin	1.85 ± 0.02	2.06 ± 0.06	0.56 ± 0.01	2.60 ± 0.08	0.90 ± 0.04	1.80 ± 0.03	4.4 ± 0.2
Cassanidin A	3.7 ± 0.1	2.10 ± 0.09	0.83 ± 0.01	4.3 ± 0.1	1.34 ± 0.04	2.16 ± 0.09	6.3 ± 0.2
(Epi)-guibourtinidol-(epi)-catechin or isomer 1	42 ± 1	29.3 ± 0.3	17.1 ± 0.9	39.5 ± 0.4	25 ± 1	28.0 ± 0.4	47 ± 1
(Epi)-guibourtinidol-(epi)-afzelechin or isomer 1	41 ± 2	28.9 ± 0.4	18 ± 1	64 ± 2	28.1 ± 0.5	42 ± 1	81.5 ± 0.2
(Epi)-guibourtinidol-(epi)-catechin or isomer 2	4.1 ± 0.3	2.8 ± 0.2	ND	3.9 ± 0.1	0.94 ± 0.02	2.58 ± 0.04	6.6 ± 0.3
(Epi)-guibourtinidol-(epi)-afzelechin or isomer 2	ND	ND	0.47 ± 0.06	ND	ND	ND	ND
(Epi)-guibourtinidol-(epi)-afzelechin or isomer 3	25 ± 1	20.9 ± 0.5	5.9 ± 0.2	23.7 ± 0.4	11.8 ± 0.4	12.9 ± 0.4	29.4 ± 0.9
(Epi)-guibourtinidol-(epi)-catechin or isomer 3	5.8 ± 0.3	3.3 ± 0.1	0.65 ± 0.03	5.5 ± 0.2	1.30 ± 0.02	3.44 ± 0.08	9.0 ± 0.5
(Epi)-guibourtinidol-(epi)-afzelechin or isomer 4	2.8 ± 0.3	2.1 ± 0.1	0.95 ± 0.04	3.7 ± 0.2	1.4 ± 0.1	ND	4.3 ± 0.1
Catechin-guibourtinidol-cassiaflavan	3.6 ± 0.1	3.1 ± 0.1	0.75 ± 0.01	4.63 ± 0.09	1.37 ± 0.02	2.9 ± 0.2	7.1 ± 0.4
**Total proanthocyanidins**	138 ± 5	107 ± 1	48 ± 3	161 ± 2	74.2 ± 0.9	103 ± 2	206 ± 3
**Total polyphenols**	262 ± 4	236 ± 1	87 ± 4	266 ± 3	136 ± 1	200 ± 2	327 ± 5

ND, not quantified.

**Table 6:** Concentration of phenolic compounds in PLE extracts, ordered by families and expressed in mg compound/g extract (X ± SD).

	PLE 8	PLE 9	PLE 10	PLE 11	PLE 12	PLE 13	PLE 14
**Hydroxybenzoic Acids**							
Galloyl glucoside	15.4 ± 0.5	14.6 ± 0.9	20 ± 1	17 ± 1	8.6 ± 0.4	18 ± 2	2.97 ± 0.07
Galloyl glucoside derivative	2.5 ± 0.2	ND	ND	3.1 ± 0.2	ND	ND	ND
**Total hydroxybenzoic acids**	17.9 ± 0.4	14.6 ± 0.9	20 ± 1	20 ± 1	8.6 ± 0.4	18 ± 2	2.97 ± 0.07
**Flavanols**							
Theaflavin derivative	0.74 ± 0.04	0.93 ± 0.02	1.48 ± 0.01	0.69 ± 0.01	0.41 ± 0.01	0.84 ± 0.03	0.08 ± 0.01
Catechin	1.49 ± 0.08	1.69 ± 0.02	ND	4.3 ± 0.3	2.5 ± 0.3	ND	0.82 ± 0.02
(Epi)-catechin	9.2 ± 0.4	8.5 ± 0.3	10.0 ± 0.3	7.0 ± 0.5	2.2 ± 0.2	7.1 ± 0.9	1.05 ± 0.06
(Epi)-afzelechin or isomer 1	3.8 ± 0.2	4.4 ± 0.2	3.5 ± 0.2	4.5 ± 0.3	2.31 ± 0.07	3.1 ± 0.2	0.9 ± 0.1
(Epi)-afzelechin or isomer 2	ND	ND	ND	1.14 ± 0.05	0.86 ± 0.02	ND	ND
Diflavanoid or isomer 1	17.4 ± 0.7	16.6 ± 0.3	16.9 ± 0.6	10.5 ± 0.7	4.0 ± 0.1	10.0 ± 0.2	1.95 ± 0.07
Diflavanoid or isomer 2	11.5 ± 0.2	10.7 ± 0.2	12.1 ± 0.1	5.7 ± 0.2	2.06 ± 0.08	9.2 ± 0.2	1.19 ± 0.04
Diflavanoid or isomer 3	2.7 ± 0.1	2.74 ± 0.09	1.61 ± 0.08	2.1 ± 0.1	1.7 ± 0.1	1.00 ± 0.04	0.67 ± 0.02
Diflavanoid or isomer 4	0.75 ± 0.02	0.78 ± 0.03	0.49 ± 0.02	0.57 ± 0.02	0.35 ± 0.02	ND	0.14 ± 0.02
Diflavanoid or isomer 5	2.9 ± 0.1	3.03 ± 0.06	3.6 ± 0.1	1.48 ± 0.03	0.76 ± 0.05	1.59 ± 0.08	0.22 ± 0.01
**Total flavanols**	51 ± 1	49.4 ± 0.4	49 ± 1	38 ± 2	17.2 ± 0.8	33 ± 1	7.05 ± 0.01
**Flavonols**							
Astilbin	0.79 ± 0.03	0.718 ± 0.005	0.66 ± 0.03	0.54 ± 0.05	0.10 ± 0.01	0.54 ± 0.06	0.040 ± 0.004
Quercentin-3-glucoside	2.24 ± 0.1	2.11 ± 0.02	0.81 ± 0.05	1.71 ± 0.02	0.91 ± 0.02	1.8 ± 0.2	0.36 ± 0.03
Quercetin-rhamnoside	2.29 ± 0.04	2.24 ± 0.03	1.74 ± 0.02	1.59 ± 0.08	0.63 ± 0.04	2.0 ± 0.2	0.27 ± 0.01
Kaempferol-rhamnoside or isomer 1	ND	ND	3.6 ± 0.2	1.35 ± 0.01	0.89 ± 0.02	1.75 ± 0.06	0.32 ± 0.01
Kaempferol-rhamnoside or isomer 2	1.58 ± 0.03	1.42 ± 0.02	4.3 ± 0.2	1.52 ± 0.04	1.06 ± 0.03	2.46 ± 0.03	0.37 ± 0.01
**Total flavonols**	6.9 ± 0.1	6.488 ± 0.005	11.1 ± 0.5	6.72 ± 0.06	3.59 ± 0.09	8.6 ± 0.5	1.36 ± 0.06
**Flavanones**							
Pinocembrin 7-neohesperidoside	16.2 ± 0.2	16.0 ± 0.4	29.7 ± 0.3	18.0 ± 0.4	13.0 ± 0.3	21 ± 2	3.40 ± 0.08
Pinocembrin 7-rutinoside	22.0 ± 0.3	21.6 ± 0.2	46 ± 3	21.5 ± 0.8	16.0 ± 0.3	26 ± 2	4.9 ± 0.2
**Total flavanones**	38.2 ± 0.5	37.6 ± 0.5	75 ± 3	40 ± 1	29.0 ± 0.7	47 ± 5	8.3 ± 0.2
**Flavones**							
Hexametoxyflavone	2.09 ± 0.06	2.1 ± 0.1	28.1 ± 0.8	2.26 ± 0.09	2.64 ± 0.04	3.0 ± 0.2	3.0 ± 0.2
**Total flavones**	2.09 ± 0.06	2.1 ± 0.1	28.1 ± 0.8	2.26 ± 0.09	2.64 ± 0.04	3.0 ± 0.2	3.0 ± 0.2
**Proanthocyanidins/Prodelphinidins**							
(Epi)gallocatechin-(epi)catechin or isomer 1	1.83 ± 0.08	1.85 ± 0.05	4.0 ± 0.2	2.2 ± 0.1	1.20 ± 0.04	3.0 ± 0.2	0.381 ± 0.007
(Epi)gallocatechin-(epi)catechin or isomer 2	4.3 ± 0.2	4.49 ± 0.09	7.4 ± 0.4	4.8 ± 0.3	2.63 ± 0.09	5.2 ± 0.4	0.80 ± 0.02
Procyanidin derivative	1.63 ± 0.06	1.59 ± 0.04	2.8 ± 0.2	ND	2.8 ± 0.2	1.50 ± 0.05	ND
(Epi)catechin-(epi)catechin	3.2 ± 0.1	3.17 ± 0.06	3.173 ± 0.06	0.86 ± 0.02	ND	1.9 ± 0.1	ND
Cassanidin A	5.0 ± 0.2	5.1 ± 0.1	2.95 ± 0.09	1.85 ± 0.06	0.77 ± 0.04	3.32 ± 0.02	0.45 ± 0.01
(Epi)-guibourtinidol-(epi)-catechin or isomer 1	47 ± 1	41.8 ± 0.1	38.7 ± 0.5	29.7 ± 0.8	14.7 ± 0.2	44 ± 2	6.3 ± 0.4
(Epi)-guibourtinidol-(epi)-afzelechin or isomer 1	67.5 ± 0.6	71.1 ± 0.6	65 ± 1	43 ± 3	22.1 ± 0.5	61 ± 2	10.8 ± 0.3
(Epi)-guibourtinidol-(epi)-catechin or isomer 2	5.0 ± 0.2	4.75 ± 0.07	4.4 ± 0.1	2.1 ± 0.1	0.83 ± 0.04	3.6 ± 0.2	0.45 ± 0.01
(Epi)-guibourtinidol-(epi)-afzelechin or isomer 2	ND	ND	ND	ND	1.33 ± 0.03	ND	ND
(Epi)-guibourtinidol-(epi)-afzelechin or isomer 3	25.1 ± 0.2	18.3 ± 0.3	25.1 ± 0.5	18.0 ± 0.5	5.7 ± 0.3	25.9 ± 0.3	3.74 ± 0.05
(Epi)-guibourtinidol-(epi)-catechin or isomer 3	6.8 ± 0.2	6.3 ± 0.2	5.3 ± 0.2	5.3 ± 0.2	ND	5.3 ± 0.3	ND
(Epi)-guibourtinidol-(epi)-afzelechin or isomer 4	3.66 ± 0.03	3.8 ± 0.2	ND	2.9 ± 0.1	2.2 ± 0.2	2.04 ± 0.09	0.77 ± 0.02
Catechin-guibourtinidol-cassiaflavan	1.14 ± 0.03	1.16 ± 0.05	1.316 ± 0.009	ND	0.46 ± 0.02	0.51 ± 0.05	ND
(Epi)gallocatechin-(epi)catechin or isomer 1	5.5 ± 0.1	5.4 ± 0.2	4.8 ± 0.2	2.19 ± 0.08	0.83 ± 0.03	3.27 ± 0.04	0.532 ± 0.008
**Total proanthocyanidins**	171 ± 3	169 ± 1	164 ± 2	110 ± 4	53.5 ± 0.6	160 ± 5	24.1 ± 0.7
**Total pplyphenols**	286 ± 5	279 ± 2	349 ± 2	217 ± 7	114 ± 2	271 ± 12	46.8 ± 0.6

ND not quantified.

**Table 7:** Variance analysis of the proposed experimental model.

**Y_1_**					
**Variable**	**Sum of Squares**	**d.f.**	**Mean Square**	**F-Ratio**	***p*-Value**
X_1_:Temperature	252.032	1	252.032	236.47	0.0299
X_2_:EtOH	4.353	1	4.353	4.08	0.2925
X_3_:Extration time	10.6513	1	10.6513	9.99	0.1950
X_1_X_1_	22.2878	1	22.2878	20.01	0.1371
X_1_X_2_	4.92866	1	4.92866	4.62	0.2771
X_1_X_3_	6.20363	1	6.20363	5.82	0.2502
X_2_X_2_	668.137	1	668.137	626.89	0.0254
X_2_X_3_	0.946107	1	0.946107	0.89	0.5189
X_3_X_3_	28.7276	1	28.7276	26.95	0.1211
Lack-of-fit	72.3088	6	24.1029	22.61	0.1938
Pure error	1.0658	1	1.0658		
Total (corr.)	1469.87	13			
*R* ^2^	0.95008				
**Y_2_**					
**Variable**	**Sum of Squares**	**d.f.**	**Mean Square**	**F-Ratio**	***p*-Value**
X_1_:Temperature	31,277.3	1	31,277.3	1177.03	0.0173
X_2_:EtOH	24,795.9	1	24,795.9	933.12	0.0196
X_3_:Extration time	399.097	1	399.097	15.02	0.1704
X_1_X_1_	31,802.3	1	31,802.3	1196.79	0.0185
X_1_X_2_	984.21	1	984.21	37.04	0.1037
X_1_X_3_	16,413.9	1	16,413.9	617.69	0.0236
X_2_X_2_	11,195.3	1	11,195.3	421.30	0.0324
X_2_X_3_	47.0042	1	47.0042	1.77	0.4104
X_3_X_3_	2415.8	1	2415.8	90.91	0.0533
Lack-of-fit	3833.34	6	1277.78	48.09	0.1231
Pure error	26.5731	1	26.5731		
Total (corr.)	109,034	13			
*R* ^2^	0.96569				

Extraction yield (Y_1_) and total phenolic compounds (Y_2_).

**Table 8:** Theoretical values of independent variable to maximize the response variables provided by the model.

Factors	Temperature X_1_ (°C)	EtOH X_2_ (%)	Time X_3_ (min)	Theoretical Optimum
Variable Response				
Yield	200	49.8	22	34.4%
TPC	46.3	73.8	22	363 mg/g
Multiple response	146.5	54.8	3	Yield = 25.7% TPC = 281 mg/g

TPC: total phenolic compounds mg/g extract.

## Data Availability

All the data generated by this research have been included in the article. Anyway, for any assitance it is possible to contact with the corresponding author.

## Supplementary Materials

The following are available online at https://www.mdpi.com/2304-8158/10/2/398/s1, Figure S1: MS spectra of the proposed compounds, including the peak numbers of Table 2 (Peaks 1–24). Figure S2: MS spectra of the proposed compounds, including the peak numbers of Table 2 (Peaks 25–47).
