# Supplementary material for: Development of an Innovative Pressurized Liquid Extraction Procedure by Response Surface Methodology to Recover Bioactive Compounds from Carao Tree Seeds
Source: Foods. 2021 Feb 11;10(2):398. doi: 10.3390/foods10020398 (PMC7917923; doi:10.3390/foods10020398)
Supplement: Supplementary file 1 [file foods-10-00398-s001.pdf]

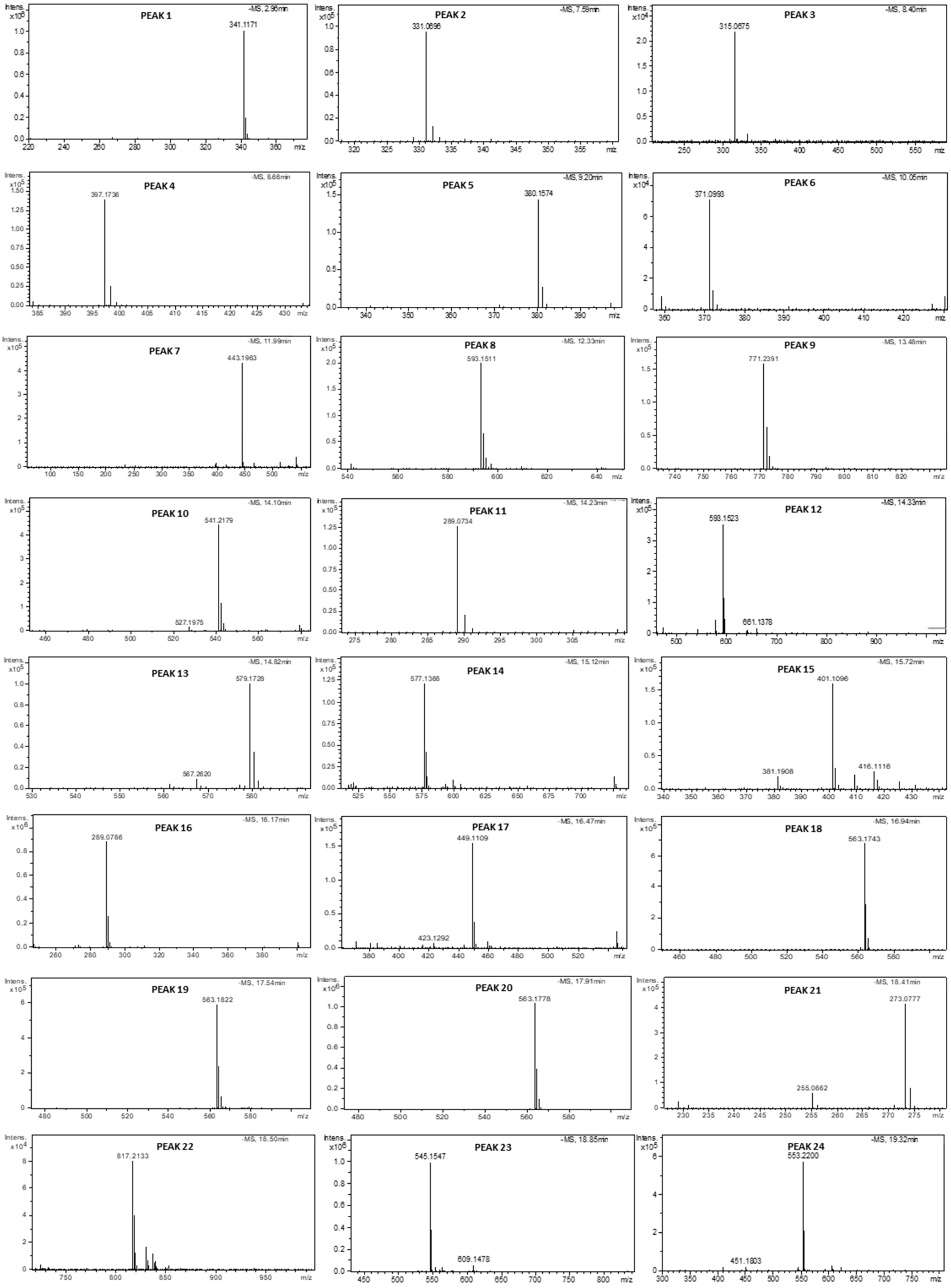


**Figure S1.** MS spectra of the proposed compounds, including the peak numbers of Table 2 (Peaks 1–24).

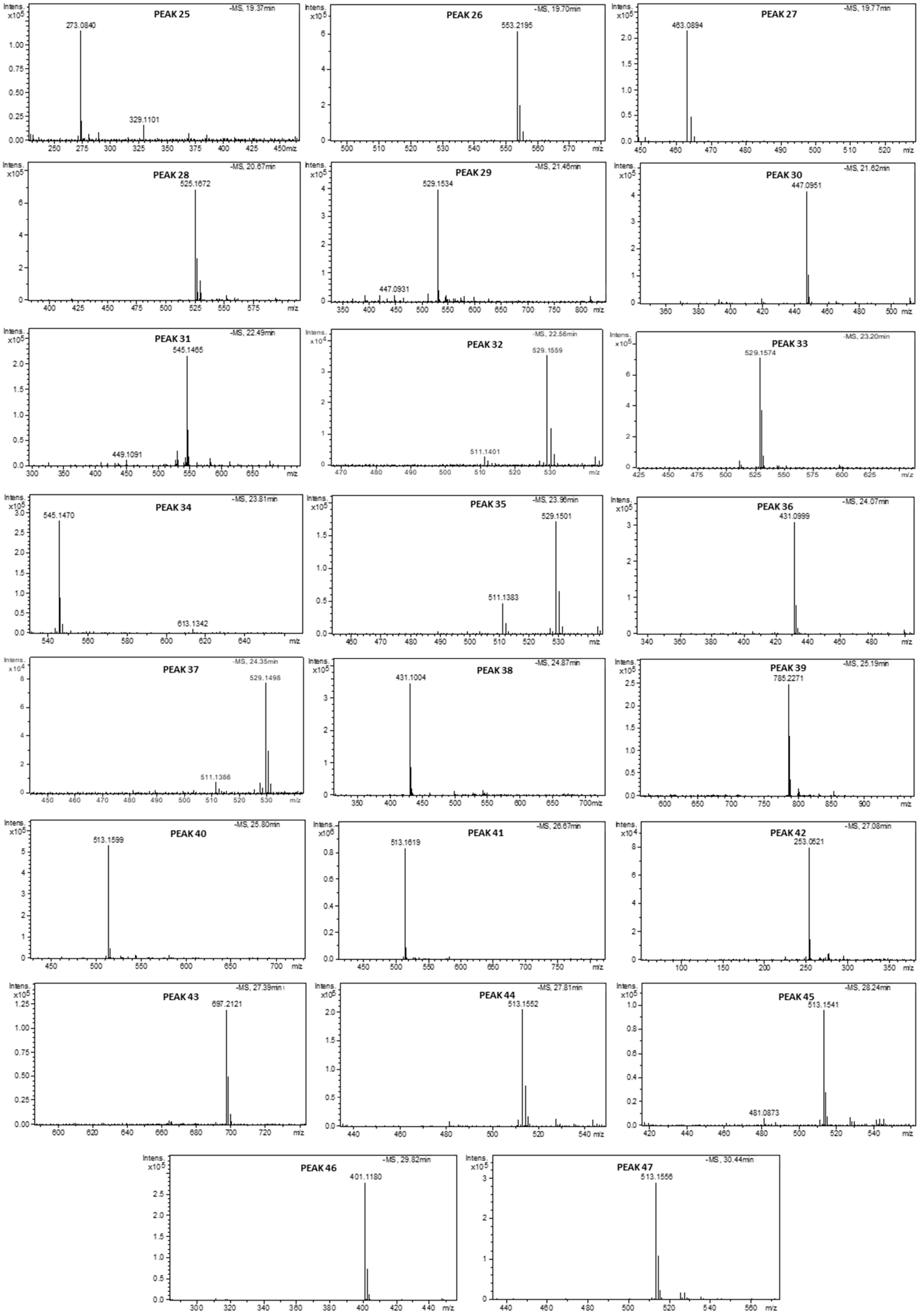


**Figure S2.** MS spectra of the proposed compounds, including the peak numbers of Table 2(Peaks 25–47).
